# *53BP1* Sensitizes Breast Cancer Cells to 5-Fluorouracil

**DOI:** 10.1371/journal.pone.0074928

**Published:** 2013-09-06

**Authors:** Xiaoyan Li, Xiaoli Kong, Xiangnan Kong, Yang Wang, Shi Yan, Qifeng Yang

**Affiliations:** 1 Department of Breast Surgery, Qilu Hospital, Shandong University, Jinan, Shandong, People's Republic of China; 2 Department of Obstetrics and Gynecology, Qilu Hospital, Shandong University, Jinan, Shandong, People's Republic of China; H.Lee Moffitt Cancer Center & Research Institute, United States of America

## Abstract

Chemoresistance of breast cancer is a worldwide problem for breast cancer and the resistance to chemotherapeutic agents frequently led to the subsequent recurrence and metastasis. In our previous study, we have found that *53BP1* showed a gradual decrease during the progression of breast cancer and loss of *53BP1* was associated with metastasis and poor prognosis in breast cancer. Here we aimed to reveal whether *53BP1* could sensitize breast cancer to 5-Fu. We found that ectopic expression of *53BP1* can significantly sensitize breast cancer cells to 5-Fu while knockdown of *53BP1* conferred the resistance. The in vivo experiments confirmed that overexpression of *53BP1* in combination with 5-Fu markedly inhibited growth of xenotransplanted tumors in nude mice when compared to either agent alone. Furthermore, we demonstrated that *53BP1* regulated the sensitivity to 5-Fu through thymidylate synthase (TS) and dihydropyrimidine dehydrogenase (DPYD). The present studies provide a new clue that combination of 5-Fu and *53BP1* could be a potential novel targeted strategy for overcoming breast cancer chemoresistance.

## Introduction

Breast cancer has been estimated to be one of the most commonly diagnosed types of female malignancy around the world. Although mortality rates of breast cancer seem to reduce during the past two decades, incidence rates continue to increase recently [1] and it is estimated about 39,510 women will die of breast cancer in the U.S. in 2012 [2]. Breast cancer is one kind of solid tumors which are sensitive to chemotherapy, thus chemotherapy is an important component in treatment of breast cancer. However chemoresistance is a worldwide problem for breast cancer and the resistance to chemotherapeutic agents frequently led to the subsequent recurrence and metastasis of cancer. Until now, the detailed mechanisms involved in chemoresistance are still largely unknown. Therefore, it is in urgent need to search for novel markers that could predict the response to chemotherapy.

5-Fluorouracil (5-Fu) plays an important role in standard chemotherapy protocols for a variety of solid tumors including breast cancer. But it is limited in clinical application due to the resistance. 5-Fu is antimetabolite inhibitors of de novo purine and pyrimidines syntheses and it is converted intracellular into 5′-fluoro-2′-deoxyuridine by thymidine phosphorylase. Subsequently it is phosphorylated by thymidine kinase into 5-fluoro-2′-deoxyuridine 5′-monophosphate (FdUMP). FdUMP which is the active form of 5-Fu inhibits thymidylate synthase (TS) so as to inhibit DNA synthesis. In addition, 5-FU can be converted into fluoro-5,6-dihydrouracil (FUH2), the inactive form of 5-Fu, by dihydropyrimidine dehydrogenase (DPYD) to lose its function [3]. Also TS and DPYD are reported to be predictive markers for 5-FU in cancers [4], [5]. Therefore the expression and activity of TS and DPYD are two major factors in molecular signaling pathway of chemoresistance to 5-Fu.

Human *53BP1* (p53 Binding Protein 1) was first identified by Iwabuchi et al. [6] and it was mapped to chromosomes 15q15–21 [7]. *53BP1* has been reported to be a candidate tumor suppressor by many studies [8]–[11]. Our collaborative groups have revealed that tumors with lower *53BP1* had significant poor metastasis free survival. [12]. Our previous studies also have demonstrated that *53BP1* showed a gradual decreased protein levels during the progression of breast cancer and it had lower expression in cancer lesions than in the matched non-tumor lesions. In addition *53BP1* could inhibit cell proliferation and invasiveness of breast cancer through nuclear factor-kappaB pathway [13]. All the above data raise the question whether *53BP1* has the effect on 5-Fu treatment of breast cancer.

In the present study, we aimed to reveal the potential role of *53BP1* in response to 5-Fu and provide a new clue for future clinical treatments of breast cancer patients who are resistant to 5-Fu treatment.

## Materials and Methods

### Cell culture and transfection

Breast cancer cell lines MCF-7, MDA-MB-231, MDA-MB-468, and T47D were obtained from American Type Culture Collection (ATCC, Rockville, MD, USA). They were routinely cultured in appropriate medium supplemented with 10% FBS and 100 units of penicillin-streptomycin at 37°C with 5% CO2 in a humidified incubator. The plasmids were constructed and the cells were transfected as previously described [13], [14].

### Reagents

Antibody against P21, Bax, Histone H2AX, TS and DPYD were purchased from Cell Signaling Technology (Beverly, MA, USA). Antibody against Bcl-2 was from Dako (Carpinteria, CA, USA). Rabbit anti-53BP1 antibody was from Bethyl Laboratories (Montgomery, USA). Signal silence TS siRNA, DPYD siRNA and their control siRNA were purchase from Cell Signaling Technology. Other reagents were from Sigma-Aldrich (St. Louis, MO, USA) unless specifically described.

### Western blot analysis

Cells were lysed with radio immunoprecipitation assay (RIPA) buffer (Shennengbocai, Shanghai, China) with protease inhibitors. Equal amount of protein were loaded on a SDS-PAGE gel and transferred to polyvinylidene fluoride membranes (ImmobilonP; Millipore, Bedford, MA). The membrane was incubated with the corresponding primary antibodies overnight at 4°C after blocking with 5% non-fat milk for 1 hour. Subsequently the secondary antibody was added for 1 hour at room temperature. Signals were detected using enhanced chemiluminescence. β-actin was used as the loading control.

### Quantitative reverse-transcription PCR analysis (qRT-PCR)

RNA was extracted with TRIZOL reagents (TaKaRa, Dalian, China). Briefly, total 0.8 µg RNA was reverse transcribed to cDNA by PrimerScript RT Reagent Kit (TaKaRa, Dalian, China). qRT-PCR was performed with Applied Biosystems StepOne and StepOnePlus Real-Time PCR Systems. GAPDH was used as an endogenous loading control. Relative quantification analysis was performed using the comparative C (T) (2^(−ΔΔCT)^) method. All the experiments were repeated at least three times.

### Cell viability assay

The cell viability was determined by 3-(4, 5-dimethylthiazol-2-yl)-2, 5-diphenyl tetrazolium (MTT) assay as described earlier [15] and the relative cell viability percentage is expressed as (absorbance of treated wells/absorbance of untreated wells).

### Immunofluorescence staining

The experiments were performed as described previously [16]. The cells were grown on coverslips in the 24-well plates, and then the cells were fixed with 4% paraformaldehyde and permeabilized using 0.1% Triton-X100 in PBS (PBST). Cells were blocked with rabbit anti-53BP1 and anti-H2AX antibody and hodamine-conjugated anti-rabbit secondary antibody. The cells were further stained with 4, 6-diami-dino-2-pheny-lindole (DAPI).

### Cell cycle analysis by flow cytometry

The cell cycle analysis were done as previously described [15]. Cells were harvested and washed by PBS and then stained with 50 mg/ml propidium iodide for 30 min. For fluorescence-activated cell sorting (FACS) analysis, data was collected using a FACSCalibur flow cytometer (Becton Dickinson, Franklin Lakes, NJ, USA).

### In vivo experiments

The in vivo experiments were approved by the Animal Care and Use Committee of Shandong University. They were performed as described previously [13]. Briefly, five million cells were injected subcutaneously into each flank of BALB/c nu/nu mice. Then 5-Fu was injected by tail vein three times per week for two weeks at a dose of 30 mg/kg. Tumor diameter was measured with calipers and the tumor volume was calculated by the formula: (width)^2^ × length/2. When mice were sacrificed, the formed breast cancer tumors were embedded in paraffin after being fixed in 10% formalin. Then the slides from the paraffin were subjected to terminal nucleotidyl transferase–mediated nick end labeling (TUNEL) assay (Beoytime, Beijing, China).

### Statistical analysis

The software SPSS 18.0 (Chicago, IL, USA) was used to analyze the data. Student's t test was used to determine significance between two groups. All experiments were repeated at least three times. The results were shown as mean ± standard deviation (SD). The results were considered statistically significant when P value<0.05.

## Results

### High expression of *53BP1* was correlated with 5-Fu sensitivity in breast cancer cell lines

**Figure 1:**
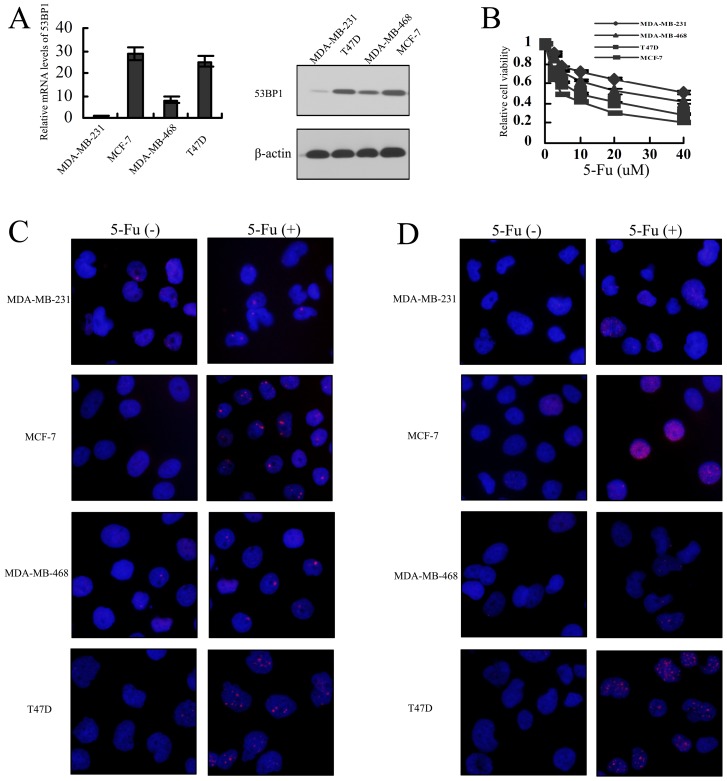
Expression of *53BP1* correlates with 5-Fu sensitivity in breast cancer cell lines. (A) Expression mRNA levels and protein levels of *53BP1* in breast cancer cell lines. GAPDH or actin was used as the endogenous control. (B) The sensitivity of breast cell lines to 5-Fu were tested with MTT assay. The data were expressed as mean ± SD. (C) The localization of *53BP1* in breast cancer before and after the treatment of 5-Fu (5 **uM**). The immunofluorescent images of cells were stained with *53BP1* antibody. The nuclear staining with Nuclei was counterstained with DAPI. Red, *53BP1*; Blue, DAPI. (D) The localization of H2AX in breast cancer before and after the treatment of 5-Fu. The immunofluorescent images of cells were stained with H2AX antibody. The nuclear staining with Nuclei was counterstained with DAPI. Red, H2AX; Blue, DAPI.

In order to explore the effect of *53BP1* on 5-Fu in breast cancer, we first detect the association between *53BP1* expression and 5-Fu sensitivity in 4 breast cancer cell lines. As shown in Fig. 1A and 1B, MDA-MB-231 and MDA-MB-468 cells with lower levels of *53BP1* was more resistant to 5-Fu while T47D and MCF-7 with higher levels of *53BP1* was sensitive to 5-FU. We also detected the the localization of *53BP1* and H2AX foci in breast cancer before and after the treatment of 5-Fu. As shown in Figure 1C and 1D, we found that the MDA-MB-231 and MDA-MB-468 cells had fewer both *53BP1* and H2AX foci than T47D and MCF-7 cells. These results suggested that relative expression of *53BP1* may correlate with 5-Fu sensitivity in breast cancer.

### *53BP1* sensitizes breast cancer cells to 5-fluorouracil

**Figure 2:**
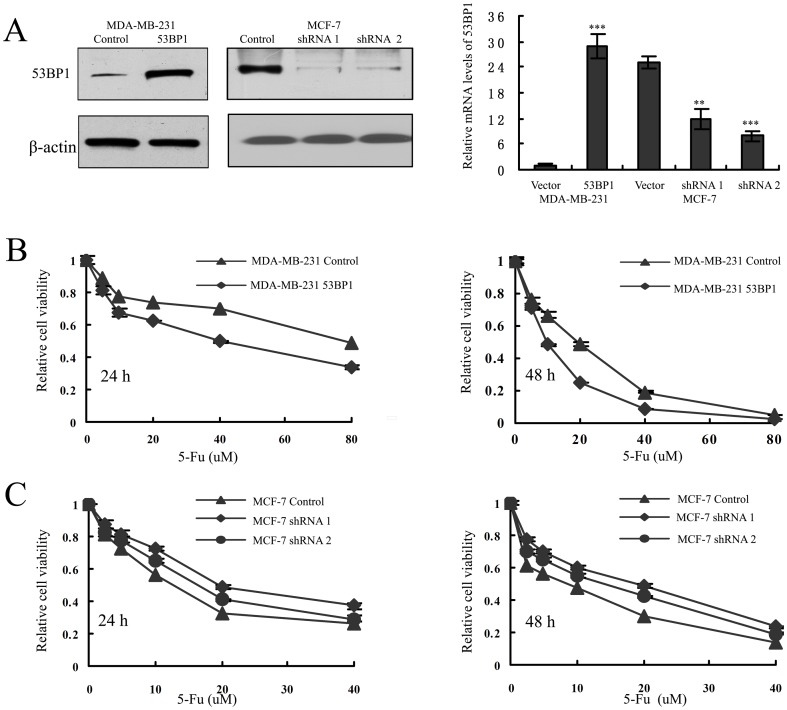
*53BP1* sensitizes breast cancer cells to 5-Fu in vitro. (A) The transfection efficiency was measured by western blot analysis (left) and qRT-PCR (right). Results are shown for one of the three independent experiments performed. **p<0.01, ***p<0.001. (B) MDA-MB-231 control and *53BP1* overexpressing cells were treated with 5-Fu for 24 h (left) or 48 h (right). The effect of *53BP1* on 5-Fu sensitivity was measured by MTT assay. (C) MCF-7 control and *53BP1* knockdown cells were treated with 5-Fu for 24 h (left) or 48 h (right). The effect of *53BP1* on 5-Fu sensitivity was measured by MTT assay. The relative cell viability percentage is expressed as (absorbance of treated wells/absorbance of untreated wells).

To evaluate the role of *53BP1* conferring sensitivity to 5-Fu in breast cancer cells, we established stable cell lines that overexpressing *53BP1* in MDA-MB-231 and stable MCF-7 cell lines with shRNA-mediated knockdown of *53BP1*. The transfection efficiency was confirmed by western blot and quantitative reverse-transcription PCR analysis (Figure 2A).

To investigate whether *53BP1* could modulate 5-Fu sensitivity in the above transfected breast cancer cells, we first performed a MTT assay as described earlier [17]. As shown in Figure 2B, MDA-MB-231 cells overexpressing *53BP1* showed enhanced sensitivity to 5-Fu in a time and dosage dependent manner. Consistent with this, we found that it was more resistant to 5-Fu in *53BP1* knockdown MCF-7 cells than its control MCF-7 cells (Figure 2C). The half maximal inhibitory concentration IC50 assessed by the MTT assay was shown in Table 1.

**Table 1:** Effect of 5-Fu on cell viability assessed by the MTT method (uM).

	IC50 (mean ± sd)	
	24 h	48 h
MDA-MB-231 Control	94.37±2.68	14.89±2.12
MDA-MB-231 *53BP1*	34.74±1.57	9.40±1.75
MCF-7 Control	12.03±1.49	6.16±1.58
MCF-7 shRNA 1	22.33±1.52	13.94±2.08
MCF-7 shRNA 2	17.83±1.25	11.05±1.69

IC50 is the half maximal inhibitory concentration. The IC50 ± sd were calculated based on the results of three independent experiments.

**Figure 3:**
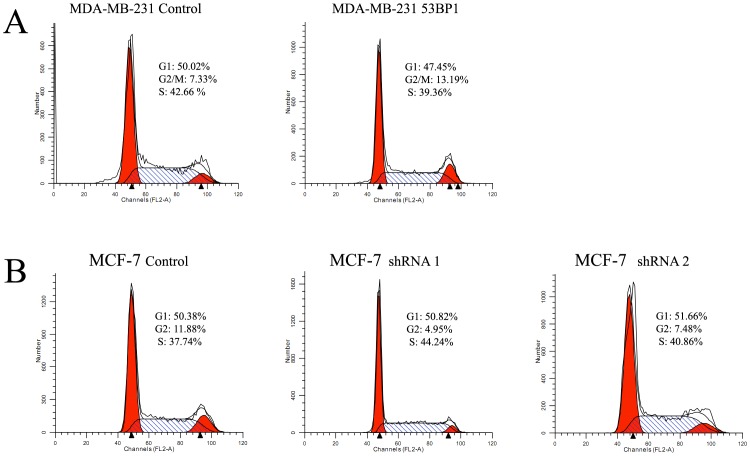
*53BP1* induced G2/M cell cycle arrest in breast cancer cells by flow cytometry analysis. (A) The effect of *53BP1* overexpression on cell cycle in MDA-MB-231 cells. (B) The effect of *53BP1* knockdown on cell cycle in MCF-7 cells.

In addition, we detected the effect on cell cycle of breast cancer cell lines. As shown in Figure 3, *53BP1* could induce the G2/M arrest in both MDA-MB-231 and MCF-7 cells. These results were inconsistent with our findings in ovarian cancer [18]. In our previous studies [13], we have found *53BP1* could inhibit the growth of breast cancer cells. Here we also examined the effects of *53BP1* on doubling time (Dt) in these cells. We found that the Dt for MDA-MB-231 was 9.6 h while the MDA-MB-231 *53BP1* cells were 14.4 h. In MCF-7 cells, the Dt for *53BP1* shRNA 1 and shRNA 2 were 13.9 h and 16.8 h respectively while the Dt was 22.8 h for control cell. This maybe one reason that *53BP1* contributes to the sensitivity to 5-Fu.

### *53BP1* sensitizes 5-FU-induced apoptosis in breast cancer cells

**Figure 4:**
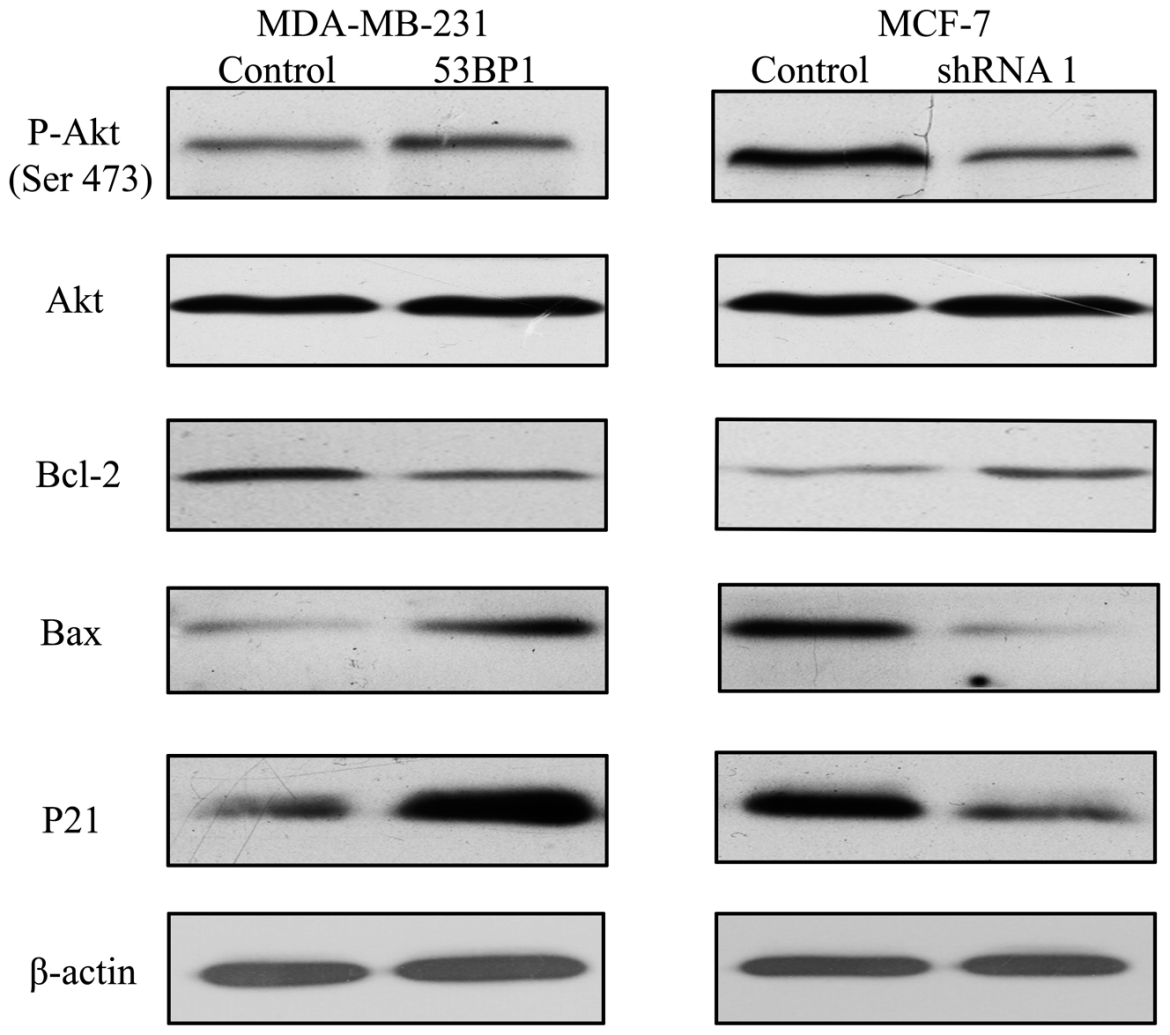
*53BP1* sensitizes 5-Fu-induced apoptosis in breast cancer cells. The apoptosis related markers Akt, p-Akt (Ser 473), Bcl-2, Bax and P21 were detected by western blot in *53BP1* transfected MDA-MB-231 and MCF-7 cells. Results are shown for one of the three independent experiments performed.

As 5-Fu could inhibit cancer cell growth and induce apoptosis, so we detected the cell apoptosis associated protein in *53BP1* transfected breast cancer cells. As shown in Figure 4, the results from western bolt analysis were also in concordance with that of the MTT assay in MDA-MB-231 and MCF-7 cells. The levels of p21 and Bax were increased and Bcl-2 was decreased in *53BP1* transfected MDA-MB-231 cells. Also knockdown of *53BP1* dramatically decreased p21 and Bax and increased the Bcl-2 level.

### *53BP1* sensitizes breast cancer cells to 5-fluorouracil in vivo

**Figure 5:**
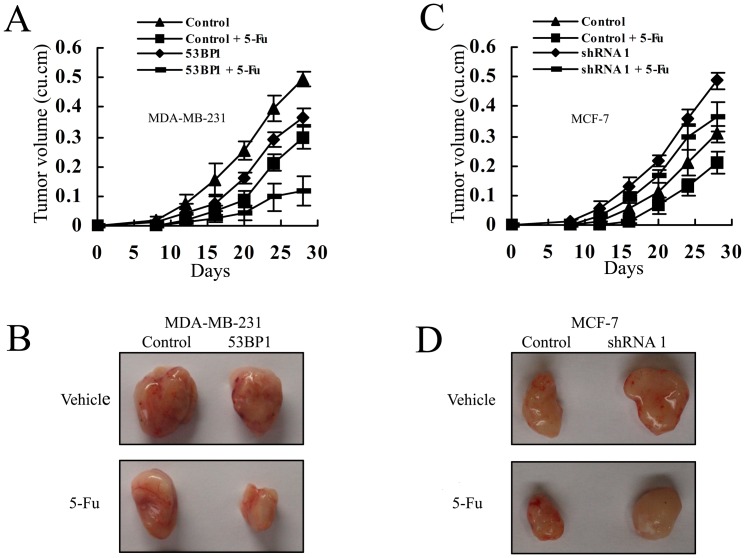
*53BP1* sensitizes breast cancer to 5-Fu in vivo. (A) Growth curves of mammary tumors for control and *53BP1* overexpressing MDA-MB-231 cells with 5-Fu or vehicle. (B) Growth curves of tumors for control and *53BP1* knockdown MCF-7 cells with 5-Fu or vehicle. Tumor diameter was measured with calipers and the tumor volume was calculated by the formula: (width)^2^ × length/2. Each group consisted of seven mice. Error bars represent ± SD. (C) Representative tumors isolated from mice after implantation with control or *53BP1* over expressing MDA-MB-231 cells and treated with 5-Fu or vehicle as described in panel A. (D) Representative tumors isolated from mice after implantation with control or *53BP1* knockdown MCF-7 cells and treated with 5-Fu or vehicle as described in panel B.

We next investigate the effect of *53BP1* on 5-Fu sensitivity in vivo using nude mice xenograft studies. MDA-MB-231 cells overexpressing *53BP1*, MCF-7 cells with *53BP1* knockdown, and their respective control cells were injected subcutaneously into each flank of female nude mice. After establishment of tumors for about a week, 5-Fu was injected by tail vein three times per week for two weeks at a dose of 30 mg/kg. Then the mice were maintained for another three weeks. As shown in Figure 5A and 5B, while overexpressing of *53BP1* or 5-Fu treatment alone significantly inhibited tumor growth, combination of upregulation *53BP1* and 5-Fu treatment resulted in an additive inhibition of tumor growth versus either treatment alone. The similar results were observed in *53BP1* knockdown MCF-7 cells. While the control MCF-7 cells treated with 5-Fu significantly inhibited the tumor growth, there was no significant different in the *53BP1* knockdown MCF-7 cells with or without 5-Fu administration (Figure 5C and 5D). All the above findings suggested that *53BP1* could sensitize breast cancer cells to 5-Fu in vivo.

**Figure 6:**
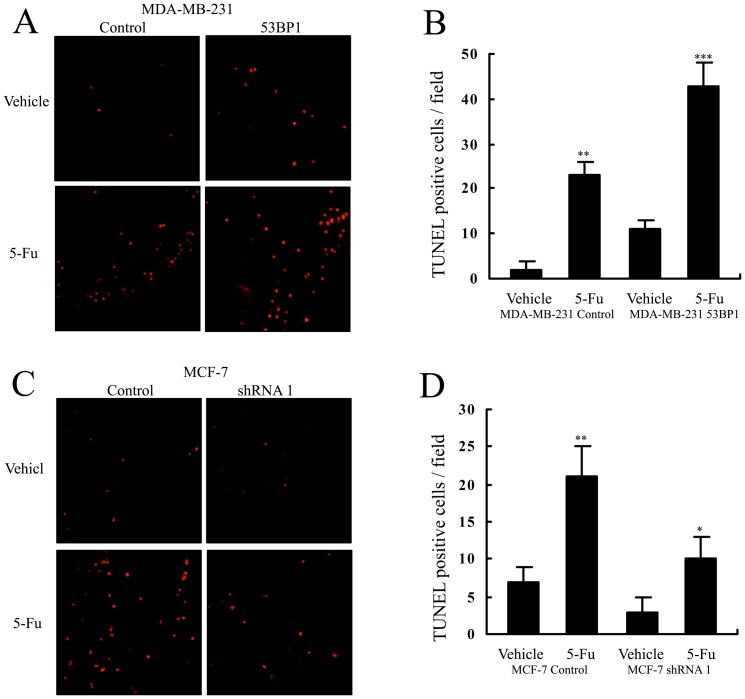
*53BP1* sensitizes 5-Fu-induced apoptosis in vivo. (A) Representative TUNEL positive staining (red fluorescence) for control and *53BP1* overexpressing MDA-MB-231 xenografts. (B) The summary graph for (A). (C) Representative TUNEL positive staining for control and *53BP1* knockdown MCF-7 xenografts. (D) The summary graph for (B). *P<0.05; **P<0.01, ***P<0.001.

Apoptosis induced by 5-Fu in tumor tissues from the vivo experiments was also measured by a terminal nucleotidyl transferase–mediated nick end labeling (TUNEL) assay. As shown in Figure 6A and 6B, when the mice were treated by 5-Fu, there were more TUNEL-positive cells in tumors from *53BP1* overexpressing MDA-MB-231 cells than those of control MDA-MB-231 cells. In addition, we found that the proportation of TUNEL-positive cells were less in *53BP1* knockdown MCF-7 tumors treated with 5-Fu than that of MCF-7 control cells (Figure 6C and 6D). These above data suggested that *53BP1* maybe a novel effective sensitizer to 5-Fu treatment in breast cancer.

### *53BP1* sensitizes breast cancer cells to 5-fluorouracil through TS and DPYD

**Figure 7:**
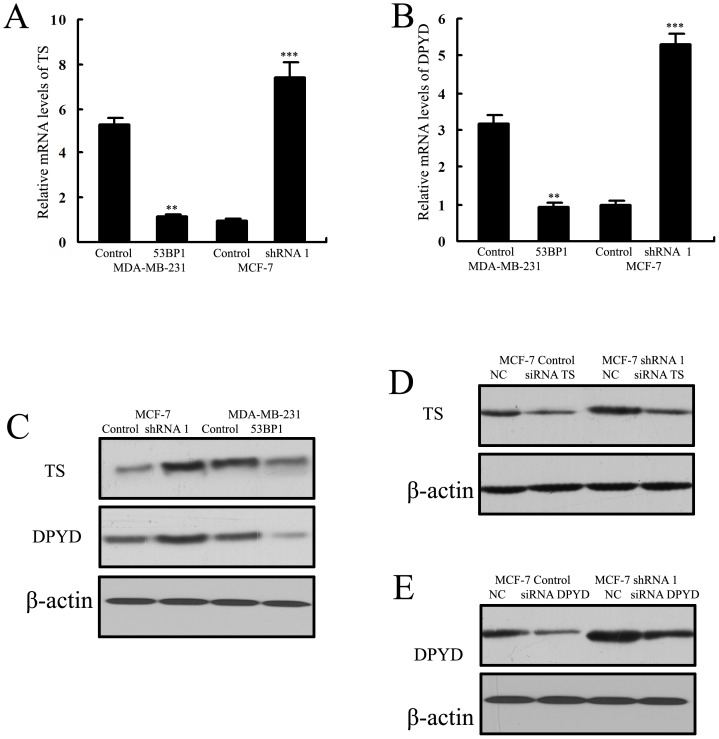
*53BP1* regulates the levels of TS and DPYD. (A) The mRNA levels of TS in *53BP1* transfected MDA-MB-231 and MCF-7 cells. (B) The mRNA levels of DPYD in *53BP1* transfected cells. (C) The protein levels of TS and DPYD in *53BP1* transfected cells. (D) The transfection efficiency of siRNA TS was confirmed by western blot analysis. (E) The transfection efficiency of siRNA DPYD was confirmed by western blot analysis. Results are shown for one of the three independent experiments performed. **p<0.01, ***p<0.001.

Several in vitro and in vivo studies have revealed a strong association between increased TS and DPYD expression and the resistance to 5-FU in various tumors including breast cancer [3], [19]–[22]. So we hypothesized that *53BP1* might contribute to 5-Fu sensitivity by regulating TS and DPYD. First we detected the mRNA and protein levels in *53BP1* transfected cells. As shown in Figure 7A, 7B and 7C, TS and DPYD protein expression levels were significantly increased in *53BP1* knockdown MCF-7 cells compared to its control cells, and they were significantly decreased in *53BP1* overexpressing MDA-MB-231 cells compared to MDA-MB-231 control cells.

To further confirm the potential role of TS and DPYD in *53BP1* mediated 5-Fu sensitivity in breast cancer cells, we used TS siRNA and DPYD siRNA to knockdown the levels of TS or DPYD in *53BP1* knockdown MCF-7 cells. The transfection efficiency was confirmed by western blot analysis (Figure 7D and 7E).

**Figure 8:**
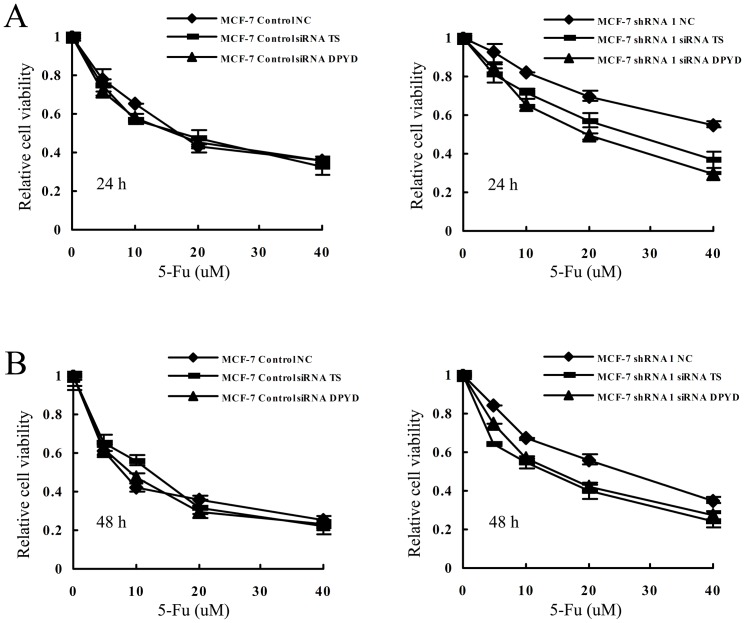
*53BP1* sensitizes breast cancer cells to 5-Fu through TS and DPYD. (A) The sensitivity of MCF-7 control and *53BP1* knockdown MCF-7 cells to 5-Fu after knockdown of TS and DPYD at the indicated time point. MCF-7 control cells, left; *53BP1* knockdown MCF-7 cells, right. (B) When the MCF-7 control and *53BP1* knockdown MCF-7 cells were treated for 48 h, the relative survival of each breast cell lines was tested with MTT assay.

Then we used MTT assay to measure the changes of sensitivity to 5-Fu with the control and TS/DPYD siRNA transfected cells. As shown in Figure 8, both TS and DPYD siRNA knockdown resulted in more sensitive to 5-Fu in *53BP1* knockdown MCF-7 cells. However no change was observed in the control MCF-7 cells after knockdown TS or DPYD. The half maximal inhibitory concentration IC50 assessed by the MTT assay was shown in Table 2. These results revealed that TS and DPYD played an important role the *53BP1* mediated sensitivity to 5-Fu in breast cancer cells.

**Table 2:** Effect of 5-Fu on cell viability assessed by the MTT method (uM).

	IC50 (mean ± sd)	
	24 h	48 h
MCF-7 Control NC	18.44±3.81	8.07±1.35
MCF-7 Control siRNA TS	16.72±2.93	10.54±2.74
MCF-7 Control siRNA DPYD	16.76±1.59	8.64±1.62
MCF-7 shRNA 1 NC	48.11±2.93	19.3±1.24
MCF-7 shRNA 1 siRNA TS	24.75±1.07	11.04±0.97
MCF-7 shRNA 1 siRNA DPYD	18.92±1.53	14.46±0.75

IC50 is the half maximal inhibitory concentration. The IC50 ± sd were calculated based on the results of three independent experiments.

## Discussion

Though many advances have been made in breast cancer systemic treatment, many patients diagnosed with early stage breast cancer eventually die of recurrent disease due to chemoresistance [23], [24]. Therefore identify novel markers that could predict the response to chemotherapy will help develop effective targeted strategies to combat breast cancer.

Recently, we and others have identified *53BP1* functioned as a novel tumor suppressor in cancers [9], [18], [25]. *53BP1* has been shown to inhibit cell proliferation, invasion, metastasis [13] and the high levels of *53BP1* were reported to be associated with better prognosis in cancers [10], [12], [26]. So this raised the hypothesis that loss of *53BP1* may contribute the treatment failure of breast cancer. However there was no evidence whether it could modulate the sensitivity of chemotherapy in breast cancers. In this manuscript we aimed to reveal the role of *53BP1* in modulating the sensitivity of breast cancers to 5-Fu.

First we investigated the relationship between *53BP1* levels and 5-Fu sensitivity in different breast cancer lines and found that high levels of *53BP1* could sensitize the response to 5-Fu. Then we used RNA interference and ectopic expression strategies to reveal the detailed function of *53BP1* in chemosensitivity. By MTT assay, we found that overexpression of *53BP1* resulted in more 5-Fu induced apoptotic cells and knockdown of *53BP1* attenuated the cell death of 5-Fu. Also *53BP1* could induce the G2/M cell cycle arrest in breast cancer cell lines. In consistent with the data, the in vivo experiments confirmed our in vitro results. We found that when the growth of tumors were inhibited by 5-Fu or overexpression of *53BP1* alone, the volumes of tumors were great significantly reduced when treated with combination of *53BP1* and 5-Fu. Tumors formed by *53BP1* knockdown MCF-7 cells became more resistant to 5-Fu while the control tumors was dramatic inhibited. The tunnel staining also demonstrated that *53BP1* could additional enhance 5-Fu induced apoptosis while downregulating of *53BP1* conferred the resistance to 5-Fu.

The development of drug resistance to 5-Fu impeded the clinical application. Previously studies have found that the effect of 5-Fu was significantly increased when TS was downregulated while the antisense downregulation of TS enhance the efficacy of 5-Fu [27]–[30]. Also the DPYD was another predictor of 5-fluorouracil toxicity reason that conferring resistance to 5-Fu [31]–[33]. Horiguchi et al [34] also reported that DPYD was a prognostic predictor in breast cancer. So we next detected if thymidine phosphorylase and dihydropyrimidine dehydrogenase were involved in the *53BP1* mediated 5-Fu sensitivity. By detecting the TS and DPYD levels in the *53BP1* transfected cell lines, we found that *53BP1* overexpression could downregulate TS and DPYD at both mRNA and protein levels while knockdown *53BP1* could uprugulate the TS and DPYD levels. In order to confirm the role of TS and DPYD in the sensitivity of 5-Fu, the siRNA TS and siRNA DPYD were used. By using siRNA TS or siRNA DPYD, we found knockdown TS and DPYD reversed the sensitivity to 5-Fu in *53BP1* knockdown MCF-7 cells. However there was no effect on the MCF-7 control cells. These findings suggested that *53BP1* sensitizes breast cancer cells to 5-fluorouracil through TS and DPYD.

In conclusion, we shows that *53BP1* can enhance sensitivity of breast cancer cell lines to 5-Fu. To the best of our knowledge, this is the first study to demonstrate the role of *53BP1* in sensitivity to 5-Fu in breast cancer. Our findings opens a new clue for enhancing the efficacy of 5-Fu in breast cancers. This provides a new concept that the combination of 5-Fu and upregulation of *53BP1* maybe a promising targeted strategy for breast cancer in the future.
